# Factors associated with subsequent surgical procedures after intramedullary nailing for tibial shaft fractures

**DOI:** 10.1007/s00590-020-02733-z

**Published:** 2020-07-12

**Authors:** Laurent A. M. Hendrickx, James Virgin, Job N. Doornberg, Gino M. M. J. Kerkhoffs, Ruurd L. Jaarsma

**Affiliations:** 1 Department of Orthopaedics and Trauma Surgery, Flinders Medical Centre, Flinders University, Adelaide, SA 5042 Australia; 2 Department of Orthopaedic Surgery, Amsterdam UMC, University of Amsterdam, Amsterdam Movement Sciences, Amsterdam, The Netherlands

**Keywords:** Tibial shaft fracture, Subsequent surgery, Predictors, Implant removal, Intramedullary nailing

## Abstract

**Introduction:**

The reported rate of subsequent surgery after intramedullary nailing (IMN) of tibial shaft fractures (TSFs) is as high as 21%. However, most studies have not included the removal of symptomatic implant in these rates. The purpose of this study was to evaluate the subsequent surgery rate after IMN of TSFs, including the removal of symptomatic implants. Secondly, this study aimed to assess what factors are associated with subsequent surgery (1) to promote fracture and wound healing and (2) for the removal of symptomatic implants.

**Methods:**

One-hundred and ninety-one patients treated with IMN for TSFs were retrospectively included. The rate of subsequent surgery was determined. Bi- and multivariable analysis was used to identify variables associated with subsequent surgery.

**Results:**

Approximately half of patients (46%) underwent at least one subsequent surgical procedure. Forty-eight (25%) underwent a subsequent surgical procedure to promote fracture or wound healing. Age (*P* < 0.01), multi-trauma (*P* < 0.01), open fracture (*P* < 0.001) and index surgery during weekdays (*P* < 0.05) were associated with these procedures. Thirty-nine patients (20%) underwent a subsequent surgical procedure for removal of symptomatic implants. There was a significantly lower rate of implant removal in ASA II (11%) and ASA III–IV (14%) patients compared to ASA I patients (29%) (*P* < 0.05).

**Conclusions:**

Patients treated with IMN for TSFs should be consented that about one-in-two patients will undergo an additional surgical procedure. Half of these procedures are required to promote wound or fracture healing; the other half are for symptomatic implant removal.

**Level of evidence:**

Therapeutic level-IV.

## Introduction

Tibial shaft fractures (TSFs) are frequently occurring injuries [1]. Intramedullary nailing (IMN) is widely considered the best treatment for these injuries because it provides good direct relative fracture stability whilst being minimally invasive with regard to surrounding soft tissue [2]. Nevertheless, for many patients IMN is only the first operation in the process of achieving satisfactory operative outcomes, with (several) additional surgical procedures often required. Current literature reports on reoperation rates after operative treatment of TSFs ranging from 14 to 36%; however, few studies have directly investigated this study question (Table 1) [3–6]. Furthermore, the majority of these studies do not include or report on removal of implant due to local pain or irritation as a secondary procedure as these are considered discretionary. In a recent review of the literature, we found the average rate of symptomatic screw removal after IMN of TSFs to be 9% [7–12]. However, from the experience at our level-1 trauma centre we believe this to be an underestimation, hypothesizing that the true rate of screw removal, and therefore the true rate of subsequent surgery, is significantly higher.

**Table 1:** Previous studies investigating factors associated with subsequent surgery after operative treatment of tibial shaft fractures

Authors	Patients	Minimum follow-up	Subsequent surgery rate	Factors associated with subsequent surgery
Stavrou et al. [3]	151 treated with IMN	12 months	21%	42B or 42C AO/OTA fracture type Alcohol abuse
Fong et al. [4]	157 treated with IMN 36 treated with plate fixation	Unclear	13.5% overall	Open fractures Transverse fractures
Bhandari et al. [5]	80 treated with IMN 108 treated with plate fixation 4 treated with external fixator	12 months	16.3% for IMN 22.4% overall	Open fractures Cortical contact < 50% Transverse fractures
Harris and Lyons [6]	124 treated with IMN 17 treated with external fixator 1 treated with plate fixation	6 months	35.8% overall	42B AO/OTA fracture type Gustilo–Anderson Grade II and III

Surgery for the removal of symptomatic implants can have a significant impact at a socio-economic level [13] and can increase the risk of additional complications [13, 14]. Decreasing the rate of these procedures should therefore be considered an important goal. However, to the best of our knowledge, thus far, no factors associated with implant removal have been identified.

The primary aim of this study was to assess the total rate of subsequent surgery after IMN of TSFs, including symptomatic implant removals. The secondary aim was to assess what patient, trauma and fracture characteristics are associated with (1) subsequent surgery for wound and fracture healing and (2) subsequent surgery for implant removal. This knowledge will allow clinicians to better inform patients on expected outcomes following surgery. Additionally, it will allow for better insight into the total health economic costs associated with IMN of TSFs.

## Materials and methods

### Ethics

In accordance with the Declaration of Helsinki, our institutional review board granted approval for this study (Reference number: AUD/19/SAC/250).

### Study design, setting and participants

As per protocol, all TSFs at our level-1 trauma centre are treated with reamed IMN with the TRIGEN Intramedullary Nailing System (Smith & Nephew, Andover, MA USA) with proximal and distal interlocking screws. Postoperatively, patients were allowed to weight bear as tolerated. Patients were routinely seen at 2, 6 and 12 weeks after surgery, or longer in case of an atypical recovery. Implant removal was not part of the standard treatment.

We included all skeletally mature patients with traumatic TSFs who were treated with IMN between January 2009 and September 2016, allowing for a minimum follow-up of 2.5 years. Patients treated for pathological fractures, patients with incomplete records and patients with inadequate follow-up (i.e. < 12 weeks) were excluded.

### Variables, outcome measures, data sources and bias

Two authors not involved in patient care (LH and JV) assessed radiographs, patients’ files, operation reports and CT scans to collect patient, trauma, fracture and treatment characteristics.

Independent variables included: (1) gender; (2) age; (3) multi-trauma; (4) trauma mechanism; (5) American Society of Anesthesiologists Physical Status (ASA-PS) [15]; (6) open fracture; (7) OTA/AO type of tibial fracture; (8) location of tibial fracture; (9) presence of fibula fracture; (10) the use > 2 proximal screws; (11) the use > 2 distal screws; (12) surgery during weekend or weekday; (13) after-hours surgery; and (14) level of surgeon. Fractures were classified into three groups according to the OTA/AO Fracture and Dislocation Classification Compendium: 42A1-3, 42B1-3 and 42C1-3. Trauma mechanism was classified as either low energy (< 30 km per hour or a fall from < 3 m) or high energy. After-hours surgery was defined as any surgery starting between 18.00 pm and 08.00 am. The cut-off for > 2 proximal or distal interlocking screws was chosen because, from our experience, the use of more locking screws is usually related to surgery for more complex fractures.

The primary outcome of this study was subsequent surgery, including elective procedures. It was recorded whether patients underwent one, two or more than two subsequent surgical procedures. Subsequent surgical procedures were also categorized into the following: (1) subsequent surgery to promote union (dynamization, nail exchange, bone graft); (2) wound closure (delayed primary wound closure, skin graft, flap or closure of fasciotomy wounds); (3) fasciotomies for postoperative compartment syndrome; (4) surgery to treat infection; (5) surgery to correct malunion or rotational malalignment; (6) surgery for wound healing (washout and debridement); (7) removal of interlocking screw due to irritation or pain; and (8) removal of tibial nail due to pain or irritation. Using these eight categories, two main groups were distinguished: (1) patients with a subsequent surgical procedure for fracture and wound healing (categories 1–6) and (2) and patients with a subsequent surgical procedure to remove symptomatic screws and/or tibial nail (categories 7 and 8).

### Statistical analysis

Qualitative assessment of the data was performed. Descriptive statistics were calculated: means and standard deviations for normally distributed continuous variables, median and range for non-normally distributed continuous variables and frequencies and percentages for categorical variables.

Bivariable analysis was performed to assess whether any independent variables were associated with each respective group of subsequent surgical procedures. Binary logistic regression was used for continuous variables, and *χ*^2^ test or Fisher exact was used for categorical and ordinal variables. Variables with a *P* value < 0.1 were subsequently entered in a multivariable binary logistic regression with a stepwise backward selection procedure. At each step, the variable with the largest *P* value was eliminated. This process was repeated until all variables in the equation reached a *P* value < 0.05. Multivariable binary logistic regression was limited to five events per variable.

Regarding subsequent surgery for symptomatic screws, we performed a subgroup analysis of patients who had undergone protocolled low-dose postoperative bilateral CT scans for the assessment of rotational malalignment [16]. This protocol was implemented at our institution in 2009 with an initial adherence rate of 43%. In 2018, the adherence rate of this protocol had increased to 83%. In a previous study, we analysed these postoperative CT scans to assess the incidence of iatrogenic screw penetration in the proximal and distal tibiofibular joint [17]. In the current study, we reused these data, to assess whether these types of screw penetration are associated with a higher rate of symptomatic screw removal.

## Results

From 2009 to 2016, 251 patients were treated with IMN for TSFs. Sixty patients (24%) were excluded: 36 patients (14%) had inadequate follow-up, 21 patients (8%) were followed up externally, one patient received palliative care after surgery, one patient had incomplete records and one patient had a pathological fracture.

A total of 191 patients were included. The majority of patients were male (71.2%) with a median age of 37 years (range, 14–90 years). Eighty patients (42%) sustained the fracture in a high-energy trauma, and 39 (20%) were polytrauma patients. Further patient and fracture characteristics are displayed in Table 2.

**Table 2:** Patient demographics and fracture characteristics (*n* = 191)

*Patient characteristics*	
Age, median years (range)	37 (14–90)
Gender, *n* (%)	
Male	55 (29%)
Female	136 (71%)
Multi-trauma, *n* (%)	
No	152 (80%)
Yes	39 (20%)
Trauma mechanism, *n* (%)	
Low energy	111 (58%)
High energy	80 (42%)
ASA-PS, *n* (%)	
ASA I	92 (48%)
ASA II	64 (34%)
ASA III–IV	35 (18%)
*Fracture characteristics*	
Open fracture, *n* (%)	
No	128 (67%)
Yes	63 (33%)
AO/OTA type, *n* (%)	
42A1-3	119 (62%)
42B1-3	44 (23%)
42C1-3	28 (15%)
Location, *n* (%)	
Proximal	6 (3%)
Middle	58 (30%)
Distal	116 (61%)
Segmental	11 (6%)
Fibula fracture, *n* (%)	
No	27 (14%)
Yes	164 (86%)
*Surgery characteristics*	
> 2 proximal screws, *n* (%)	
No	175 (92%)
Yes	16 (8%)
> 2 distal screws, *n* (%)	
No	145 (76%)
Yes	46 (24%)
Day of operation, *n* (%)	
Weekday	127 (66%)
Weekend	64 (34%)
After-hours surgery, *n* (%)	
No	145 (76%)
Yes	46 (24%)
Level primary surgeon, *n* (%)	
Consultant	71 (37%)
Fellow	68 (36%)
Registrar	52 (27%)

Eighty-seven patients (46%) underwent at least one subsequent surgical procedure. The most frequent indication for a first subsequent surgical procedure was screw removal due to irritation or pain (40%), followed by closure of wounds (25%) (Table 3). Twenty-nine patients (15%) underwent at least two subsequent surgical procedures. The most frequent second additional surgical procedures were performed for wound healing (31%), followed by closure of wounds (21%) (Table 3). Thirteen patients (7%) underwent more than two additional surgical procedures.

**Table 3:** Overview of the first and second additional subsequent surgical procedures patients underwent

Type of subsequent surgical procedure	First subsequent surgical procedure, *n* (%)	Second subsequent surgical procedure, *n* (%)
Surgery to promote union	9 (10%)	3 (10%)
Surgery to close wounds	22 (25%)	6 (21%)
Fasciotomy postoperative compartment syndrome	4 (5%)	2 (7%)
Surgery to treat infection	2 (2%)	1 (3%)
Surgery to correct malunion	5 (6%)	3 (10%)
Surgery to promote wound healing	6 (7%)	9 (31%)
Removal symptomatic screw	35 (40%)	5 (17%)
Removal symptomatic nail	4 (5%)	5 (17%)
*Total*	87 (100%)	29 (100%)

### Subsequent surgery fracture and wound healing

Forty-eight patients (25%) underwent a first subsequent surgical procedure to promote fracture or wound healing. Bivariable analysis demonstrated that age (*P* < 0.05), multi-trauma (*P* < 0.001), trauma-mechanism (*P* < 0.001), open fracture (*P* < 0.01), AO/OTA type (*P* < 0.01), the use of more than 2 proximal interlocking screws (*P* < 0.05) and surgery during weekdays (*P* < 0.05) were associated with subsequent surgical procedures for fracture and wound healing (Table 4).

**Table 4:** Bivariable Analysis of Patient, Trauma, Fracture and Treatment Characteristics and Subsequent Surgery for Fracture and Wound Healing (*n* = 191)

Variable	Subsequent surgery for fracture and wound healing		*P *value
	No	Yes	
Gender, *n* (%)			0.30
Male	99 (73%)	37 (27%)	
Female	44 (80%)	11 (20%)	
Age, mean years (SD)	41.8 (17.4)	35.1 (16.8)	0.024*
Multi-trauma, *n* (%)			< 0.001*
No	123 (81%)	29 (19%)	
Yes	20 (51%)	19 (49%)	
Trauma mechanism, *n* (%)			< 0.001*
Low energy	94 (85%)	17 (15%)	
High energy	49 (61%)	31 (39%)	
ASA-PS, *n* (%)			0.36
ASA I	71 (77%)	21 (23%)	
ASA II	44 (69%)	20 (31%)	
ASA III–IV	28 (80%)	7 (20%)	
Open fracture, *n* (%)			
No	105 (82%)	23 (18%)	0.001*
Yes	38 (60%)	25 (40%)	
AO/OTA type, *n* (%)			0.003*
42A1-3	98 (82%)	21 (18%)	
42B1-3	30 (68%)	14 (32%)	
42C1-3	15 (54%)	13 (46%)	
Location, *n* (%)			0.16
Proximal	3 (50%)	3 (50%)	
Middle	43 (74%)	15 (26%)	
Distal	91 (78%)	25 (22%)	
Segmental	6 (55%)	5 (45%)	
Fibula fracture, *n* (%)			0.39
No	22 (81%)	5 (19%)	
Yes	121 (74%)	43 (26%)	
> 2 proximal screws, *n* (%)			0.017*
No	135 (77%)	40 (23%)	
Yes	8 (50%)	8 (50%)	
> 2 distal screws, *n* (%)			0.86
No	109 (75%)	36 (25%)	
Yes	34 (74%)	12 (26%)	
Day of operation, *n* (%)			0.012*
Weekday	88 (69%)	39 (31%)	
Weekend	55 (86%)	9 (14%)	
After-hours surgery, *n* (%)			0.86
No	109 (75%)	36 (25%)	
Yes	34 (74%)	12 (26%)	
Level surgeon, *n* (%)			0.68
Consultant	51 (72%)	20 (28%)	
Fellow	51 (75%)	17 (25%)	
Registrar	41 (79%)	11 (21%)	

*Binary logistic regression or *χ*^2^ test was significant at *P* < 0.05

Multivariable analysis subsequently identified younger age (*P* < 0.01), multi-trauma (*P* < 0.01), open fracture (*P* < 0.001) and surgery during weekdays (*P* < 0.05) as independent predictors (Table 5).

**Table 5:** Multivariable logistic regression analysis subsequent surgery fracture and wound healing

Variable	Odds ratio (95% confidence interval)	*P* value
Age	0.96 (0.94–0.99)^a^	< 0.01
Multi-trauma	3.20 (1.42–7.22)	< 0.01
Open fracture	4.14 (1.89–9.05)	< 0.001
Surgery on weekdays	2.96 (1.22–7.17)	0.02

^a^Odds ratio per year increase in age

### Subsequent surgery for removal of symptomatic screws and/or nail

Removal of symptomatic screws and/or nail occurred on average 578 days after the index procedure (range 94–1850 days). Thirty-nine patients (20%) underwent a first subsequent surgical procedure for removal of symptomatic screws or nails. Bivariable analysis indicated that only ASA-PS was associated with this type of subsequent surgery (Table 6). The rate of implant removal was significantly lower in ASA II and ASA III–IV patients as compared to ASA I patients (*P* < *0.05*).

**Table 6:** Bivariable analysis of patient, trauma, fracture and treatment characteristics and subsequent surgery for symptomatic screws or nail

Variable	Surgery symptomatic screws or nail		*P* value
	No	Yes	
Gender, *n* (%)			0.63
Male	107 (79%)	29 (21%)	
Female	45 (82%)	10 (18%)	
Age, mean years (SD)	41.1 (18.1)	36.1 (14.3)	0.11
Multi-trauma, *n* (%)			0.38
No	119 (78%)	33 (22%)	
Yes	33 (85%)	6 (15%)	
Trauma mechanism, *n* (%)			0.90
Low energy	88 (79%)	23 (21%)	
High energy	64 (80%)	16 (20%)	
ASA-PS, *n* (%)			0.01*
ASA I	65 (71%)	27 (29%)	
ASA II	57 (89%)	7 (11%)	
ASA III–IV	30 (86%)	5 (14%)	
Open fracture, *n* (%)			
No	97 (76%)	31 (24%)	0.06
Yes	55 (87%)	8 (13%)	
AO/OTA-type, *n* (%)			0.99
42A1-3	95 (80%)	24 (20%)	
42B1-3	35 (80%)	9 (20%)	
42C1-3	22 (79%)	6 (21%)	
Location, *n* (%)			0.55
Proximal	6 (100%)	0 (0%)	
Middle	44 (76%)	14 (24%)	
Distal	93 (80%)	23 (20%)	
Segmental	9 (82%)	2 (18%)	
Fibula fracture, *n* (%)			0.20
No	19 (70%)	8 (30%)	
Yes	133 (81%)	31 (19%)	
> 2 proximal screws, *n* (%)			0.63
No	140 (80%)	35 (20%)	
Yes	12 (75%)	4 (25%)	
> 2 distal screws, *n* (%)			0.50
No	117 (81%)	28 (19%)	
Yes	35 (76%)	11 (24%)	
Surgery in weekend, *n* (%)			0.46
No	103 (81%)	24 (19%)	
Yes	49 (77%)	15 (23%)	
After-hours surgery, *n* (%)			0.87
No	115 (79%)	30 (21%)	
Yes	37 (80%)	9 (20%)	
Level surgeon, *n* (%)			0.55
Consultant	59 (83%)	12 (17%)	
Fellow	54 (79%)	14 (21%)	
Registrar	39 (75%)	13 (25%)	

*χ^2^ test was significant at *P* < 0.05

### Subgroup analysis of screw penetration

A total of 123 patients had undergone a low-dose postoperative CT scan to assess malalignment according to hospital protocol. Of these patients, 18 were excluded; in three patients, it was unclear which screw had been removed since no follow-up radiology was available; three patients had undergone dynamization to promote union; and in 12 patients, the tibial nail had been revised, removed or exchanged after the CT scan. In the remaining 105 patients, no association between proximal or distal tibiofibular screw penetration and screw removal could be demonstrated (Table 7).

**Table 7:** Bivariable analysis of tibiofibular screw penetration and subsequent surgery for screw removal

Tibiofibular screw penetration	Total	Screw removal		*P* value
		Yes	No	
Proximal, *n* (%)				
No screw penetration	61	7 (11%)	54 (89%)	0.51
Screw penetration	44	7 (16%)	37 (84%)	
Distal, *n* (%)				
No screw penetration	63	10 (16%)	53 (84%)	0.57
Screw penetration	42	5 (12%)	37 (88%)	

## Discussion

Patients treated with IMN for TSFs should be consented that about one-in-two patients will undergo an additional surgical procedure. Approximately half of these additional surgical procedures are performed to promote fracture or wound healing. Age, multi-trauma, open fractures and index surgery during weekdays are predictors of this type of additional surgical procedures. The other half of procedures are discretionary: performed to remove interlocking screws and/or tibial nails causing pain or irritation. This type of procedures is less frequently performed in patients with higher ASA-PS and is not associated with tibiofibular screw penetration. These data support the consent of patients with TSFs: that IMN may not be a quick fix.

The findings of this study must be appreciated with an understanding of its limitations. Firstly, a substantial number (22%) of patients had to be excluded due to inadequate follow-up. Although loss to follow-up is a well-known problem in Orthopaedic Trauma [18]; in the current study, this relative high number was partly caused by 8% of patients being followed up externally. This is common practice at our hospital, as our hospital services rural locations more than 1000 km away. Secondly, this study was conducted retrospectively. One of the disadvantages of this design is that the independent variables tested were limited to those that had been collected previously. Potentially important variables such as alcohol abuse [3] could therefore not be included. Thirdly, this study was conducted at a single, level-1 trauma centre. This may have resulted in a slight overrepresentation of high-energy trauma and open fractures. However, since mono-trauma cases are also part of our daily routine practice and represented 80% of the entire cohort, we believe the current series is a good representation of the entire spectrum of tibial shaft fractures. Lastly, limited by the number of events per variable we were forced to group a number of independent variables. This may have concealed the effect of certain variables such as the previously documented effect of transverse fractures on the re-operation rate [4, 5].

The one-in-two reoperation rate (46%) identified in this study is substantially higher than previously reported (14–36%) [3–6]. This is mainly due to the large number (*n* = 39) of surgical procedures carried out for symptomatic screw removal, which is not included in the majority of the previously reported studies, but very important in informed consent for our patients in the overall picture. It could be argued that the removal of symptomatic locking screws is a relatively minor surgical procedure; however, from a patients’ perspective any type of surgery is often subjectively considered as major. With an estimated total procedural cost of $2000–2500 (AUD) at our institution, this type of surgery can furthermore have significant impact at a socio-economic level [13]. We therefore believe that it is important for clinicians and patients to be aware of this substantial number. It is important to note that the high rate of implant removal is not exclusive to IMN. In a randomized controlled trial comparing IMN and plate fixation of distal TSFs, the rate of subsequent surgery for implant removal was similar between both groups [19].

It is well known that open TSFs are at a higher risk of infection and non-union [20–23]. In the current study, open fractures were identified as an independent predictor of subsequent surgery. This is in line with what several previous studies have demonstrated [4–6] (Table 1). Both younger age and multi-trauma were also predictive of subsequent surgery for wound and fracture healing. Both of these variables may be considered indicative of injury severity. With regard to age, this can be explained by the bimodal distribution of TSFs: in younger patients, they are more often caused by traffic accidents, whereas in elderly patients they are most commonly caused by simple falls [24]. Lastly, surgery on weekdays was an independent predictor of subsequent surgery for wound and fracture healing. When initiating this study, we hypothesized the opposite to be true, as various studies have suggested outcome may be worse if patients are admitted or undergo surgery during the weekend [25–27]. A possible explanation for our finding could be that there may be a tendency to postpone non-acute, yet complex cases during the weekend to weekdays.

Only one variable was associated with subsequent discretionary surgery to remove symptomatic screws and/or nails: in patients with higher ASA-PS significantly less surgery was performed to remove implant. This is likely explained by surgeons and anaesthesiologists being more cautious with additional surgery in this patient group, rather than there being a causal relationship between ASA-PS and symptomatic screws. On the other hand, it may also indicate that we need to more critically review whether removal of screws and/or nails in patients with an ASA I status is necessary as one could argue that this is elective. Whilst it is suggested that screw penetration in the proximal and distal tibiofibular joint may lead to respective lateral sided knee-pain [17, 28] and lateral sided ankle pain [17], there was no higher rate of removal of these screws in our study. Future studies should aim to assess whether screw penetration in the proximal or distal tibiofibular joint indeed causes pain or affects functional outcome. It is important to note that the interlocking screws which were used in this study have been modified in order to give the screw heads a lower profile. This modification was introduced in our hospital after our final inclusion. Future studies should be performed to assess whether this modification results in lower rates of screw removal.

Although we identified several predictors for subsequent surgery for fracture and wound healing, it remains difficult to extrapolate these findings to the individual patient: we present average results of an ‘extrapolated study population’. Moreover, these predictors have not been validated [29]. In orthopaedic surgery, various studies have recently been published that use a streamlined method for developing, validating and deploying prediction models [30, 31]. The use of machine learning algorithms in these studies furthermore may allow for identifying nonlinear relations between variables [32]. Applying such methods could potentially aid in developing, validating and deploying a more practical prediction model to estimate the risk of subsequent surgery in individual patients with TSFs. This may require larger datasets and could be subject of future studies in our era of personalized care.

The current study could not identify any causal predictors of subsequent surgery for removal of implant. This might mostly be determined by type of implant used and local experiences and protocol. Given the high rate of these surgeries, future studies should aim to assess whether there are any other variables associated with these procedures. Identifying such variables may help modifying treatment in order to decrease the rate of these procedures.

In conclusion, nearly one-in-two patients treated with IMN for TSFs will undergo an additional surgical procedure. Approximately half of these procedures are required for wound and fracture healing, whilst the remaining half are discretionarily performed to remove symptomatic screws or nails. Age, open fractures and multi-trauma were independent predictors of the former, whilst a higher rate of symptomatic implant removal was seen in ASA I patients.

## Data Availability

Not applicable.
